# Explaining Differences Between Sibling Relationships in Schizophrenia and Nonclinical Sibling Relationships

**DOI:** 10.3389/fpsyt.2020.00321

**Published:** 2020-04-17

**Authors:** Léa Plessis, Hélène Wilquin, Jean-Baptiste Pavani, Evelyne Bouteyre

**Affiliations:** ^1^ Clinical Psychology, Pysychopathology and Psychoanalysis Laboratory (LPCPP), Aix Marseille Univ, Aix-en-Provence, France; ^2^ Centre for Research on the Psychology of Cognition, Language and Emotion (PsyCLE), Aix Marseille Univ, Aix-en-Provence, France

**Keywords:** sibling relationship, schizophrenia, symptom severity, adult siblings, family structure

## Abstract

**Background:**

Good sibling relationships in adulthood are known to be a protective factor for mental health. The quality of these relationships is influence by the sibship’s inherent characteristics (e.g., birth order, number of brothers and sisters, sex composition, age gaps). The present study explored whether these same determinants can help to explain how individuals experience their relationship with a sibling who has been diagnosed with schizophrenia.

**Method:**

A total of 374 adults completed the Adult Sibling Relationship Questionnaire, a scale that probes the quality of these relationships on three dimensions: warmth, conflict, and rivalry. We also collected sociodemographic data and information about family structure from each of the participants. Participants were divided into two matched groups: nonclinical sibling group (*n* = 187) or schizophrenia sibling group (*n* = 187). Regression analyses were conducted to extract possible predictors of sibling relationship quality for each group. Further regression analyses then focused exclusively on relationships with an ill sibling, in order to study the role of disease-related variables in explaining each of the three dimensions.

**Results:**

Results showed that sociodemographic and family structure data explained a significant proportion of variance in the sibling relationship, but solely for nonclinical siblings. When participants had a sibling with schizophrenia, we found that disease-related variables (symptom severity, frequency of treatment) also had to be included to predict the conflict dimension.

**Conclusions:**

Our results suggest that feelings of conflict experienced by the schizophrenia sibling group were fueled by the symptoms the ill person displayed. Healthy brothers and sisters probably have only a poor understanding of these symptoms. This could be improved by supporting them and helping them learn more about the disease. Future research will have to prove that providing such support for siblings does indeed improve the quality of their sibling relationships and, by so doing, enhance the wellbeing of both members of a sibling dyad.

## Introduction

In recent years, the move toward deinstitutionalizing psychiatry has led to considerable interest in how families function as a system. Owing to ever shorter hospital stays, patients soon go back to living in the community or, more often than not, with their families (1). As a result, the latter are now regarded as the pillars of the care pathway for patients with schizophrenia (SZ) (2). Indeed, family members are referred to in the scientific literature as *caregivers* (3–5). Nevertheless, research on patients’ families all too often turns out to be research on parents, if not just mothers (3, 6, 7).

Although brothers and sisters are rarely include in these studies, they are certainly not spared the shock caused by the onset of a psychiatric disorder (8). Indeed, they are often in the front line when the very first signs of the disease appear. Moreover, unlike their parents, they are not shielded from this adversity by a generational barrier (9). Disease onset may therefore affect the sibling bond and threaten the benefits that are assumed to flow from a good sibling relationship, such as better mental health and social, cognitive, and emotional development (10, 11).

Sibling relationships are one of the longest lasting types of relationships we can have (12, 13), and are constantly adapting and changing. More specifically, in adulthood, they can be described along three dimensions: warmth, conflict, and rivalry (14, 15). *Warmth* can be defined as perceived closeness and support among siblings. *Conflict* refers to disagreements and quarrels within the relationship, while *rivalry* refers to competition between siblings for their parents’ love and attention. Up to adolescence, a good sibling relationship is characterized by great warmth, scant conflict, and low rivalry (16), and can be an important protective factor for mental health (17). In adulthood, sibling relationships are generally more elective and therefore more peaceful (less warmth, but also less conflict and rivalry) than they are in childhood (18–21).

Each sibling relationship is unique, shaped by a shared history and the particular composition of the sibling and family groups. Nevertheless, the variance in these sibling relationships can be partly explained by various inherent characteristics of the sibship, including birth order (22), number of brothers and sisters (15, 21), sex composition (15, 21), ages (15, 19), and age gaps (15).

*Sex composition* has received considerable attention from researchers seeking to explain disparities in the experience of sibling relationships. In adulthood, brothers and sisters of opposite sexes describe less conflict in their relationships than same-sex siblings do (15). Women report more conflict and rivalry in their sibling relationships than men do. However, they also describe greater warmth (18) and more satisfaction with these relationships (23–25). Research results confirm that relationships between sisters are more intense than relationships between brothers (21, 26).

Studies about sibling birth order and its impact on children’s personality are controversial (27). Researchers nevertheless agree that birth order plays a part in children’s cognitive development and caregivers’ behaviors toward siblings (28). Firstborn siblings tend to display more educational behaviors and take care of their younger siblings (23, 29–32). Longitudinal research needs to be undertaken to confirm the link between birth order and prosocial behavior among adult siblings. This research has already been done with children, but birth order is generally overlooked in the exploration of adult sibling relationships.

Comparisons between siblings with a small *age gap* can also induce a form of rivalry in adulthood (33), despite the fact that inequalities recede over time. Whereas Riggio (34) observed greater warmth between adult siblings when they were next to each other in the birth order (34), Stocker, Lanthier, and Furman found less conflict between siblings who were farther apart in age (15). These results suggest that siblings who are closer in age have a more intense experience of the sibling relationship (more warmth, more conflict, and more rivalry).

The *number of brothers and sisters* can also influence the number of sibling alliances and rivalries. For example, Stocker et al. (1997) found that in adulthood, larger sibships report more rivalry and less warmth than smaller ones (15). The authors interpreted this result in the light of early relationships, arguing that children in large families are acutely aware of their parents’ limited attentional resources as they are growing up, and remain particularly sensitive to potential disparities in the amount of parental attention they receive (15).

These variables therefore seem to have an impact on adults’ experience of their sibling relationships. But do they exert the same influence when one of the siblings has been diagnosed with SZ?

Onset of SZ symptoms generally occurs between the ages of 15 and 25 years. At this age, siblings are often still living under the same roof. Moreover, unlike the relatives of individuals with developmental disorders or intellectual disability, the family and friends of persons with SZ generally knew them prior to symptom onset. In addition to having to manage their negative emotional responses (35, 36), they therefore have to revise their future plans for and with their ill sibling (37). Living with a person with SZ is a strange and bewildering experience (38). The symptoms (i.e., delusions, hallucinations, behavioral problems, anhedonia, cognitive disorders, and isolation) and the chronic nature of the disorder place a heavy burden on all family members (39).

Although authors have become increasingly interested in the experience of healthy individuals who have a sibling with SZ, there is still scant research on these very particular sibling relationships. Most have focused either on the healthy brother or sister’s coping strategies (40–43), the emotional experience of younger children (35, 36), or their involvement as caregivers to their ill sibling (44–49). To our knowledge, healthy adults’ experience of their relationship with a sibling diagnosed with SZ has only been explored in two studies.

The more recent study looked at how the aggressive behaviors toward self or others displayed by persons with first episode psychosis (FEP) affect their relationships with their healthy brothers and sisters (50). Participants completed the Adult Sibling Relationship Questionnaire (ASRQ), a self-report questionnaire measuring the three dimensions (warmth, conflict, and rivalry) of sibling relationships. Results indicated that these aggressive behaviors (taking drugs, history of violence, and suicide attempts) are detrimental to the sibling relationship. We can nevertheless observe that in this study only 1.6% of individuals who had experienced FEP displayed aggressive behaviors toward others; the most common aggressive behaviors were self-harm behaviors such as drug taking or suicide attempts. These results, albeit interesting, concerned quite young siblings (*M*_age_ = 21.7, *SD* = 4.4). The earlier study, by contrast, looked at the determinants of the SZ sibling relationship in adulthood (51). More specifically, the authors assessed the effect of eight variables on healthy adults’ experience of the sibling relationship: family cohesion during childhood, threats or physical violence, fear of the ill sibling, personal gains from coping with the latter’s illness, ability of the ill sibling to control the symptoms, being a parent or not, being in a female or nonfemale dyad, and SZ symptoms reported by the caregivers themselves. Respondents reported a better relationship when they had grown up in a more cohesive family environment and when they experienced more personal gains from coping with the challenges of their sibling’s mental illness. By contrast, believing that the sibling with SZ could control his or her symptoms had a negative impact on the sibling relationship. In conclusion, the results of these two studies on relationships between healthy adults and siblings with SZ point in the same direction (50, 51), indicating that negative assessments of these relationships arise mainly from fear, especially when the target sibling is violent or displays threatening behavior (50, 51).

Following on from these studies, the present research further explored the determinants of relationships between healthy adults and siblings with SZ. For the first time, however, we ran comparisons with a matched nonclinical sibling group, formed especially for this study. We postulated that whereas these nonclinical sibling relationships can be explained—at least in part and as shown in the literature review above—by various characteristics inherent to the sibship (e.g., family structure characteristics such as age gap between siblings, sex composition, number of brothers and sisters, etc.), this is less the case for relationships between healthy adults and siblings with SZ. More specifically, based on the findings of the two above-mentioned studies (50, 51), we suggested that SZ-related variables (particularly the severity with which symptoms are perceived by the healthy sibling) must also be taken into account to better explain the three dimensions (warmth, conflict, and rivalry) of relationships between healthy adults and siblings with SZ. The short version of the ASRQ was used to measure these three dimensions of adult sibling relationships.

## Materials and Methods

### Participants

#### Two Initial Groups

We recruited two initial groups of French volunteers who had all (*N* = 1645) completed an online questionnaire: a large group of 1,444 adults with nonclinical siblings (76.5% female; *M*_age_ = 25.91 years, *SD* = 8.31) drawn from the general population between September and November 2017; and a smaller group of 201 adults (77.1% female; *M*_age_ = 37.9 years, *SD* = 12.08) with a sibling with a SZ between November 2017 and February 2018. The inclusion criteria for all participants were (a) aged at least 18 years, and (b) at least one sibling aged 18 years or over. For participants in the SZ sibling group, there was a third criterion: having a sibling who had been diagnosed with SZ by a healthcare professional.

#### Matching Procedure

The matching procedure was performed manually, using the group of siblings of individuals with SZ as a reference (*n* = 201). Each participant in the SZ sibling group was matched with a participant in the nonclinical sibling group on four criteria: age (within 5 years), sex, sex of target sibling, and birth order (younger or older than target sibling). When several participants had the same profile, the matching was carried out randomly.

#### Two Matched Groups Siblings With and Without SZ

With these four criteria, we were able to match 187 participants in the SZ sibling group with 187 siblings from the general population. We therefore ended up with two matched groups, each with 187 participants (*N* = 374). In each group, the mean age was 35.9 years (*SD* = 10.7), 46.5% of participants were younger than the target sibling, 49.7% were older, and 3.7% were twins. In both groups, 77.5% of participants were women answering about their relationship with a brother (80.7%). Fourteen participants in the initial group of 201 individuals with a sibling diagnosed with SZ could not be paired, mostly because of their age: nine of them were aged over 64 years, whereas the oldest participant in the nonclinical group was 64 years old. The remaining five were twins for whom we were unable to find a matching twin of the same sex in the initial nonclinical group (*n* = 1444).

### Procedures

Janghorban, Roudsari, and Taghipour recommended recruiting participants *via* social media, instead of relying solely on psychiatric institutions or charitable bodies, in order to reach a broader population (52). Participants were therefore recruited *via* social media (support groups for the relatives of individuals with mental disorders) and through the Union Nationale de Familles ou Amis de Personnes Malades et/ou Handicapées Psychiques^1^ (UNAFAM), a French charity that offers support to the families of persons with chronic mental disorders.

An electronic link to an online form was sent by social media group administrators, organization websites, and charity newsletters to potential participants. After reading an information letter, participants had to validate their consent online (53). They could then gain access to a self-administered online questionnaire that took approximately 40 min to complete.

### Measures

#### Sociodemographic and Family Structure Data

We collected participants’ sociodemographic data, physical proximity data, and information about their family structure and looked for differences between the two groups. The sociodemographic category includes sex, age, and socio-economic status variables. The physical proximity category includes residence and visit frequency variables. The family structure category includes sibling size, sex of target sibling, age difference between the two siblings, birth order, and birth order of target sibling variables.

#### Clinical Data About the Target Sibling With SZ

For the SZ sibling group only, we collected clinical information about the ill sibling through a questionnaire that we specifically designed for the purposes of the present study. Participants completed a 10-item questionnaire about the pathology of their sibling diagnosed with SZ (e.g., age at symptom onset, year of diagnosis, type of SZ (paranoid, hebephrenic, etc.), perceived degree of severity, number of hospitalizations, type and frequency of professional follow-up, primary caregiver, and social activity of target sibling.

#### Short Version of the Adult Sibling Relationship Questionnaire (ASRQ-S)

The ASRQ-S is a self-report questionnaire assessing the qualitative features of sibling relationships in young adulthood and beyond. Participants were asked to report on a single sibling relationship. The original long-form version of the ASRQ (81 items) was developed by Stocker et al. (15) as an age-appropriate extension of the Sibling Relationship Questionnaire (54).

The short form of the ASRQ (ASRQ-S), developed by Lanthier, Stocker, and Furman but not yet validated or published, includes 47 of the 81 items in the full ASRQ. These 47 items are divided into eight subscales corresponding to the three above-mentioned factors: knowledge, intimacy, and emotional support (warmth); antagonism, dominance, and quarreling (conflict); and maternal rivalry and paternal rivalry (rivalry). We translated the ASRQ-S into French using Vallerand’s back-translation procedure, after obtaining the consent of the original authors (55). A native English bilingual translated the English version of the ASRQ-S into French, and a second bilingual translated this French version back into English. When compared, the two English versions were initially found to have substantial incongruities. The French version was therefore self-administered by 10 siblings to identify potential problems or ambiguities arising from the translation. Their responses were used to produce the final French version of the ASRQ-S.

Three of the items (items 10, 11, and 27) making up the dominance subscale were deleted from the French version because the component coefficients were not conclusive (> .30). After deleting these items, the French version of the ASRQ-S contained 44 items. It was found to have good internal consistency (minimum *α* = 0.65, maximum *α* = 0.96).

The subscales assessing maternal and paternal rivalry give participants the option of not responding if one or both parents have died. Whenever data were missing, we performed the analyses on the remaining data.

### Data Analytic Strategy

The data analysis took place over two phases, both featuring regression analyses and performed with R 3.5.0 software. The first phase was intended to ascertain whether family-structure variables were equally predictive of sibling relationship quality across the two groups. The second phase was intended to ascertain whether the quality of SZ sibling relationships was better predicted by family-structure variables or by SZ-related variables.

#### Comparisons Between Nonclinical Sibling and SZ Sibling Groups

In the first phase, we tested the hypothesis that sociodemographic variables (i.e., sex, age, socioeconomic status) and family-structure variables (i.e., sibling size, sex of the target sibling, difference in age between the two siblings in absolute values, the participant’s birth order, the target sibling’s birth order, place of residence, and visit frequency) are less predictive of the quality of the sibling relationship in the SZ sibling group.

We used two complementary methods to test this first hypothesis. The first method consisted in observing whether the percentage of the variance in each of the three dimensions of the adult sibling relationship (i.e., warmth, conflict, rivalry) explained by sociodemographic and family-structure variables was smaller in the SZ sibling group than in the nonclinical sibling group. To this end, ordinary least squares regressions were computed separately for each group. These regressions were computed in a hierarchical fashion, entering first the sociodemographic variables, then the family-structure variables as predictors. This method had the advantage of allowing the two groups to be directly compared on (a) the percentage of the explained variance, and (b) the number of statistically significant predictors. However, as the regressions were computed separately for each group, it did not allows us to determine whether putative differences between these two groups were statistically significant.

Consequently, we applied a second method to determine whether considering the moderating effect of group (SZ siblings vs. nonclinical siblings) significantly increased the model’s fit to the data. To this end, we computed two ordinary least squares regressions at the level of the whole sample. Both regressions contained the above-mentioned sociodemographic and family-structure variables as predictors. However, the first regression only contained the additive effect of group among its predictors, whereas the second model contained both its additive and interaction effects. Model comparisons allowed us to see whether the addition of this interaction effect significantly improved the fit of the model to the data. This method had the advantage of enabling us to determine whether there was a statistically significant difference in the way our predictors influenced our outcome variables according to the group.

To avoid any multicollinearity issues, we computed for each regression analysis the adjusted generalized variance inflated factor of each predictor (56, 57), and removed the predictors showing a value which was above 5 on this factor (57).

#### SZ-Related Variables Explaining the Sibling Relationship in the SZ Sibling Group

The second phase of our data analytic strategy was designed to test the hypothesis that the quality of a sibling relationship involving an individual with SZ is predicted not only by family-structure variables, but also by SZ-related variables. This phase would be particularly important if, as expected, family-structure variables proved to be less predictive of the quality of these relationships. To test this hypothesis, we examined whether considering SZ-related variables (e.g., type, severity) significantly increased the percentage of the explained variance in each of the three dimensions of the adult sibling relationship, compared with when only sociodemographic and family-structure variables were considered. Once again, we carried out ordinary least squares regressions in a hierarchical fashion, entering the sociodemographic variables first, then the family-structure variables, and lastly the SZ-related variables.

## Results

### Descriptive Statistics and Between-Groups Comparison

Descriptive statistics for the sociodemographic, physical proximity, family structure, and ASRQ-S (warmth, conflict, rivalry) data we collected on the two groups are set out in **Table 1**. The SZ sibling and nonclinical sibling groups were similar overall, as they only differed from each other on six sociodemographic and family-structure variables: the proportion of unemployed participants, which was higher in the nonclinical sibling group (χ² = 5.22, *p* < 0.05); the proportion of target siblings who were the youngest in their sibship, which was higher in the SZ sibling group (χ² = 18.71, *p* < 0.001); the proportion of target siblings who occupied an intermediate position in their sibship, which was higher in the nonclinical sibling group (χ² = 11.97, *p* < 0.001); sibling size, which was higher in the SZ sibling group (*t* = −2.01, *p* < 0.05); difference in age between the two siblings in absolute values, which was higher in the SZ sibling group (*t* = −4.28, *p* < 0.001); and mean frequency of encounters, which was higher in the SZ sibling group (*t* = −2.54, *p* < 0.05).

**Table 1:** Participants’ sociodemographic, physical proximity, and family structure characteristics.

Variables	Modality (for categorical variables)	Total sample (*N* = 374)	Nonclinical siblings (*n* = 187)	SZ siblings (*n* = 187)	*t* or chi²
Sex (%)	Female	288 (77.00)	144 (77.00)	144 (77.00)	0
Mean age in years (*SD*)		35.96 (10.75)	35.93 (10.76)	36 (10.77)	−0.07
Socioeconomic status (%)	Never worked/long-term unemployed	21 (5.67)	16 (8.70)	5 (2.67)	5.22*
	Small employer or own account worker	9 (2.43)	2 (1.09)	7 (3.74)	1.76
	Managerial/administrative/professional occupation	135 (36.39)	63 (34.24)	72 (38.50)	0.56
	Intermediate occupation	42 (11.32)	11 (5.98)	31 (16.58)	9.35**
	Lower supervisory & technical	91 (24.53)	50 (27.72)	41 (21.39)	1.68
	Semi-routine occupation	4 (1.08)	2 (1.09)	2 (1.07)	0
	Full-time student	62 (16.71)	36 (19.57)	26 (13.90)	1.75
	Retired	7 (1.89)	3 (1.63)	4 (2.14)	0
Sibling size (*SD*)	Number of siblings per family	3.17 (1.55)	3.01 (1.64)	3.33 (1.43)	−0.32*
Sex of target sibling (%)	Female	74 (19.79)	37 (19.79)	37 (19.79)	0
Absolute value of the mean age difference between the two siblings in years (*SD*)		4.29 (3.01)	3.64 (2.35)	4.94 (3.43)	−1.30*
Participant’s birth order (%)	Youngest	94 (25.13)	39 (20.86)	55 (29.41)	3.20
	Middle	131 (35.03)	67 (35.83)	64 (34.22)	0.05
	Oldest	149 (39.84)	81 (43.32)	68 (36.36)	1.61
Target sibling’s birth order (%)	Youngest	77 (20.59)	21 (11.23)	56 (29.9)	18.91***
	Middle	168 (44.92)	101 (54.01)	67 (35.83)	11.77***
	Oldest	129 (34.49)	65 (34.76)	64 (34.22)	0
Place of residence (%)	Same	19 (4.95)	7 (3.74)	12 (6.42)	0.89
Mean visit frequency, ranging from 1 (*never*) to 7 (*every day*) (*SD*)		3.06 (1.33)	2.88 (1.14)	3.23 (1.48)	−2.54*
Warmth		2.80 (0.89)	3.07 (0.94)	2.53 (0.78)	−6.04***
Conflict		1.92 (0.72)	1.84 (0.66)	1.99 (0.76)	2.11*
Rivalry		0.59 (0.52)	0.46 (0.45)	0.73 (0.55)	4.99***

The last column consist of the mean or frequency difference in the variable of interest between the non-clinical and SZ sibling groups. Student’s t test were used when this variable was numeric, and Pearson’s chi² test were used when this variable was nominal.

* p < 0.05. ** p < 0.01. *** p < 0.001.

### Regression Analyses

#### Did Sociodemographic and Family-Structure Variables Explain Adult Sibling Relationships?

##### Warmth in the Adult Sibling Relationship

Results of the hierarchical regression analyses are provided in **Tables 2** and **3** (column 1). When the sociodemographic variables were entered alone as predictor variables, the multiplicative model explained approximately the same percentage of the variance in the warmth of the sibling relationship as the additive model (i.e., 10%). Consistently, the former model did not fit the data significantly better than the later (*F*(9, 351) = 1.19, *p* > 0.05).

**Table 2:** Standardized regression coefficients of the hierarchical regression analyses for warmth, conflict, and rivalry for models with and without the interaction with the group (nonclinical sibling group and SZ sibling group).

Variable	Modalities compared for categorical variables	Warmth				Conflict				Rivalry			
		Additive		Multiplicative		Additive		Multiplicative		Additive		Multiplicative	
		Step 1	Step 2	Step 1	Step 2	Step 1	Step 2	Step 1	Step 2	Step 1	Step 2	Step 1	Step 2
Grp	SZ vs. nonclinical	−0.63***	−0.73***	−0.62	−1.50**	0.26*	0.21	−0.55	−0.61	0.54***	0.55***	−0.13	−0.05	
Sex	Male vs. female	−0.05	−0.06	−0.04	0.15	−0.10	−0.10	−0.05	−0.09	−0.32*	−0.33**	−0.32	−0.47**	
Age in years		−0.10	−0.05	−0.19*	−0.04	0.07	0.11	−0.07	−0.04	0.12	0.17*	0.20*	0.28**	
Socio-economic status	Small employer or own account worker vs. never worked/long-term unemployed	0.82*	0.52	1.36	1.59*	−1.07**	−0.86*	−1.72*	−1.85*	−0.20	−0.04	−0.05	0.07	
	Managerial/administrative/professional occupation vs. unemployed	0.25	0.04	0.23	−0.17	−0.32	−0.18	−0.47	−0.33	−0.09	0.03	−0.07	0.18	
	Intermediate occupation vs. unemployed	0.13	0	−0.10	−0.23	−0.50	−0.38	−1.33**	−1.23**	−0.10	−0.02	−0.65	−0.53	
	Lower supervisory & technical vs. unemployed	0.32	0.05	0.30	−0.23	−0.51*	−0.37	−0.77**	−0.57	−0.16	−0.04	−0.51	−0.14	
	Semi-routine occupation vs. unemployed	0.06	−0.87	−0.92	−2.13**	0.03	0.31	0.83	1.18	0.63	0.91	2.31**	3.65***	
	Fulltime student vs. unemployed	−0.04	−0.22	0.03	−0.33	−0.25	−0.24	−0.51	−0.52	0.13	0.16	0.01	0.12	
	Retired vs. unemployed	1.09	0.55	1.04	0.78	−0.31	−0.20	−0.81	−0.52	−1.16*	−0.99*	−1.54*	−1.31*	
Sibling size		0.04		0.15		−0.01		0		−0.08		−0.29**		
Sex of target sibling	Male vs. female	−0.38**		−0.67***		−0.08		−0.10		−0.10		−0.07		
Age difference between the two siblings		−0.04		0.04		0.05		−0.04		−0.02		0.06		
Birth order	Intermediate vs. youngest	0.02		0.35		−0.17		−0.39		−0.16		−0.04		
	Oldest vs. youngest	−0.14		−0.04		−0.26		−0.35		−0.21		−0.59**		
Birth order of target sibling	Intermediate vs. youngest	0.01		0.19		−0.02		0.11		0.03		0.10		
	Oldest vs. youngest	−0.08		−0.06		0.10		0.34		0.08		−0.22		
Residence	Different vs. same	0.83**		0.74**		−0.48		−0.66*		−0.40		−0.50		
Visit frequency (from 1= never to 7= every day)			0.49***		0.67***		−0.07		−0.10		−0.09		−0.20*	
Grp*sex	Male vs. female			0	−0.25			−0.13	−0.11			−0.02	0.11	
Grp*socio-economic status	Small employer or own account worker vs. never worked/long-term unemployed			−0.79	−1.24			1.30	1.82			0.36	0.42	
	Managerial/administrative/professional occupation vs. unemployed			0	0.40			0.71	0.83			0.40	0.33	
	Intermediate occupation vs. unemployed			0.25	0.35			1.63*	1.77**			1.26*	1.31*	
	Lower supervisory & technical vs. unemployed			0.02	0.51			0.97	0.98			1.11*	0.86	
	Semi-routine occupation vs. unemployed			1.91	2.64**			−1.20	−1.28			−2.96**	−4.35***	
	Fulltime student vs. unemployed			−0.23	0.19			0.81	0.96			0.65	0.59	
	Retired vs. unemployed			0	0.06			1.31	1.16			1.19	1.12	
Grp*sibling size					−0.26*				−0.04				0.33*	
Grp*sex of target sibling	Male vs. female				0.77***				−0.06				−0.23	
Grp*age difference between the two siblings					−0.12				0.10				−0.09	
Grp*birth order	Intermediate vs. youngest				−0.29				0.32				−0.12	
	Oldest vs. youngest				0.09				0.13				0.47	
Grp*birth order of target sibling	Intermediate vs. youngest				−0.11				−0.19				−0.12	
	Oldest vs. youngest				0.01				−0.30				0.23	
Grp*visit frequency (from 1= Never to 7= Every day)					−0.32**				0.01				0.18	
Adj. R²		10.08	30.29	10.50	37.75	1.60	1.99	4.66	4.12	8.56	8.97	13.70	16.03	

Adj. R² = adjusted R squared. Step 1 = regression analyses containing only sociodemographic predictor variables; Step 2 = regression analyses containing both sociodemographic and family-structure predictor variables. The interaction between residence and the group was not included in the multiplicative models because its adjusted generalized variance inflation factor suggested multicollinearity issues.

* p < 0.05. ** p < 0.01. *** p < 0.001.

**Table 3:** Beta values of the hierarchical regression analyses for warmth, conflict, and rivalry for both nonclinical sibling group and SZ sibling group.

Variable	Modalities compared for categorical variables	Warmth				Conflict				Rivalry			
		Nonclinical siblings		SZ siblings		Nonclinical siblings		SZ siblings		Nonclinical siblings		SZ siblings	
		Step 1	Step 2	Step 1	Step 2	Step 1	Step 2	Step 1	Step 2	Step 1	Step 2	Step 1	Step 2
Sex	Male vs. female	−0.04	0.15	−0.04	−0.11	−0.05	−0.10	−0.17	−0.18	−0.37*	−0.54**	−0.32	−0.34
Age in years		−0.18*	−0.04	−0.02	−0.02	−0.07	−0.06	0.14	0.18	0.23*	0.33*	0.05	0.08
Socio-economic status	Small employer or own account worker vs. never worked/long-term unemployed	1.31	1.53*	0.66	0.38	−1.85*	−2.03*	−0.39	0.13	−0.06	0.11	0.29	0.37
	Managerial/administrative/professional occupation vs. unemployed	0.22	−0.16	0.27	0.24	−0.51	−0.38	0.22	0.61	−0.08	0.23	0.31	0.39
	Intermediate occupation vs. unemployed	−0.10	−0.22	0.17	0.11	−1.43***	−1.29**	0.28	0.66	−0.75*	−0.62	0.57	0.65
	Lower supervisory & technical vs. unemployed	0.29	−0.22	0.37	0.30	−0.83**	−0.62*	0.19	0.52	−0.59*	−0.15	0.57	0.61
	Semi-routine occupation vs. unemployed	−0.88	−2.05**	1.14	0.55	0.89	1.24	−0.35	0.11	2.66***	4.22***	−0.61	−0.78
	Fulltime student vs. unemployed	0.02	−0.32	−0.23	−0.17	−0.55	−0.52	0.29	0.53	0.01	0.12	0.62	0.60
	Retired vs. unemployed	1	0.75	1.20	0.96	−0.87	−0.58	0.47	0.70	−1.77**	−1.51*	−0.33	−0.29
Sibling size			0.15		−0.12		0		−0.02		−0.35		0.04
Sex of target sibling	Male vs. female		−0.64***		0.12		−0.12		−0.16		−0.08		−0.28
Age difference between the two siblings			0.04		−0.10		0.11		0.07		0.06		−0.03
Birth order	Intermediate vs. youngest		0.34		0.06		−0.44		−0.06		−0.04		−0.15
	Oldest vs. youngest		−0.04		0.06		−0.38		−0.20		−0.67*		−0.10
Birth order of target sibling	Intermediate vs. youngest		0.18		0.10		0.10		−0.10		0.12		0
	Oldest vs. youngest		−0.06		−0.06		0.38		0.03		−0.27		0.02
Residence	Different vs. same		0.66*		0.92**		−0.29		−0.95*		−0.83*		−0.28
Visit frequency (from 1= *never* to 7= *every day*)			0.55***		0.46***		−0.06		−0.13		−0.21**		0
Adj *R*²		1.71	43.57	2	12.95	9.32	8.51	0	0	16.36	28.09	1.28	0

Adj. R² = adjusted R squared. Step 1 = regression analyses containing only sociodemographic predictor variables; Step 2 = regression analyses containing both sociodemographic and family-structure predictor variables.

*p < 0.05. **p < 0.01. ***p < 0.001.

SZ, schizophrenia.

By contrast, when family-structure variables were added to the predictor variables, a discrepancy emerged between the two models. The multiplicative model explained a more important percentage of the variance in warmth than the additive model (i.e., 38% vs. 30%), and fit the data significantly better than this additive model (*F*(17, 334) = 3.47, *p* < 0.001). In other words, the manner in which family structure and sociodemographic variables together predicted warmth in the SZ sibling group was different from the manner in which they did it in the nonclinical group.

When the multiplicative model with both sociodemographic and family structure variables as predictors was examined, four statistically significant interaction effects emerged. Specifically, the group (i.e., SZ sibling vs. nonclinical sibling) significantly interacted with visit frequency (*β* = −0.32, *p* < 0.01), the sex of the sibling (*β* = 0.77, *p* < 0.001), sibling size (*β* = −0.25, *p* < 0.05), and the difference between unemployed individuals and individuals with a semi-routine occupation (*β* = 2.64, *p* < 0.01) to predict warmth. These four statistically significant interaction effects shared a common point. In each case, belonging to the SZ sibling group reduced (i.e., made it closer to 0) the contribution of the variable of interest (i.e., visit frequency, sex of the sibling, sibling size, the difference between unemployed individuals and individuals with a semi-routine occupation) to warmth. For instance, the partial effect of visit frequency on warmth was positive and strong (*β* = 0.67, *p* < 0.001). The fact that the interactive effect of visit frequency and the group was of an opposite sign (i.e., it was negative), it meant that belonging to the SZ sibling group reduced the positive effect of visit frequency on warmth. Consistently, when sociodemographic and family structure variables were examined as predictors of warmth in one model for the nonclinical sibling group and in another model for the SZ sibling group (see **Table 3**), the model for the nonclinical sibling group explained a more important percentage of the variance of warmth than the model for the SZ sibling group (i.e., 44% vs. 13%).

Interestingly, the examination of the multiplicative model with both sociodemographic and family structure variables as predictors permitted to identify two statistically significant partial effects which did not interact with the group to predict warmth. These were the difference between unemployed individuals and Small employers or own account workers (*β* = 1.59, *p* < 0.05) and the residence place (*β* = 0.74, *p* < 0.01). In other terms, whether in the SZ sibling group or in the nonclinical sibling group, warmth tended to be stronger among Small employers or own account workers rather than unemployed individuals and individuals who do not live in the same residence as their sibling.

##### Conflict in the Adult Sibling Relationship

Results regarding conflict are provided in **Tables 2** and **3** (Column 2). When the sociodemographic variables were entered alone as predictor variables, the multiplicative model explained a more important percentage of the variance in conflict than the additive model (i.e., 5% vs. 2%), and fit the data significantly better than this additive model (*F*(9, 351) = 2.29, *p* < 0.05). Thus, the manner in which sociodemographic variables predicted conflict in the SZ sibling group was different from the manner in which they did it in the nonclinical group.

When the multiplicative model was inspected, we observed one statistically significant interaction effect. Specifically, the group (i.e., SZ sibling vs. nonclinical sibling) significantly interacted with the difference between unemployed individuals and individuals with intermediate occupation (*β* = 1.63, *p* < 0.05) to predict conflict. Once again, belonging to the SZ sibling group reduced the contribution of the variable of interest (i.e., the difference between unemployed individuals and individuals with intermediate occupation) to the aspect of the sibling relationship examined (i.e., conflict). Consistently, when sociodemographic and family structure variables were examined as predictors of conflict in one model for the nonclinical sibling group and in another model for the SZ sibling group (see **Table 3**), the model for the nonclinical sibling group explained a more important percentage of the variance of conflict than the model for the SZ sibling group (i.e., 9% vs. 0%).

The examination of the multiplicative model with sociodemographic variables as predictors also permitted to identify two statistically significant partial effects which did not interact with the group to predict conflict. These were the difference between unemployed individuals and Small employers or own account workers (β = −1.72, p < 0.05), and the difference between unemployed individuals and individuals with lower supervisory or technical occupation (β = −0.77, p < 0.01). In other terms, whether in the SZ sibling group or in the nonclinical sibling group, conflict tended to be weaker among small employers or own account workers and individuals with lower supervisory or technical occupations.

The addition of the family structure variables increased neither the multiplicative model fit to the data (*F*(17, 334) = 1.16, *p* > 0.05) nor the additive model fit to the data (*F*(9, 351) = 0.88, *p* > 0.05). Thus, differences between the SZ sibling group and the nonclinical sibling group in the way conflict was predicted by our variables of interest seemed to be restricted to sociodemographic characteristics.

##### Rivalry in the Adult Sibling Relationship

Results regarding rivalry are displayed in **Tables 2** and **3** (column 3). When the sociodemographic variables were entered alone as predictor variables, the multiplicative model explained a more important percentage of the variance in rivalry than the additive model (i.e., 14% vs. 9%), and fit the data significantly better than this additive model (*F*(9, 338) = 3.30, *p* < 0.001). Thus, the manner in which sociodemographic variables predicted rivalry in the SZ sibling group was different from the manner in which they did it in the nonclinical group.

When we examined the multiplicative model, three statistically significant interaction effect. Specifically, the group (i.e., SZ sibling vs. nonclinical sibling) significantly interacted with the difference between unemployed individuals and individuals with intermediate occupation (*β* = 1.26, *p* < 0.05), the difference between unemployed individuals and individuals with lower supervisory or technical occupations (*β* = 1.11, *p* < 0.05), and the difference between unemployed individuals and individuals with semi-routine occupation (*β* = −2.96, *p* < 0.01) to predict rivalry. Consistent with what we observed regarding warmth and conflict, belonging to the SZ sibling group reduced the contribution of the above-mentioned variables to the aspect of the sibling relationship examined (i.e., rivalry). Consistently, when sociodemographic and family structure variables were examined as predictors of rivalry in one model for the nonclinical sibling group and in another model for the SZ sibling group (see **Table 3**), the model for the nonclinical sibling group explained a more important percentage of the variance in rivalry than the model for the SZ sibling group (i.e., 16% vs. 1%).

The examination of the multiplicative model with sociodemographic variables as predictors also permitted to identify two statistically significant partial effects which did not interact with the group to predict rivalry. These were age (β = 0.20, p < 0.05), and the difference between unemployed and retired individuals (β = −1.54, p < 0.05). Put it differently, whether in the SZ sibling group or in the nonclinical sibling group, rivalry tended to be stronger among older individuals, unless they are retired.

The addition of the family structure variables increased neither the multiplicative model fit to the data (*F*(17, 321) = 1.55, *p* > 0.05) nor the additive model fit to the data (*F*(9, 338) = 1.17, *p* > 0.05). Thus, differences between the SZ sibling group and the nonclinical sibling group in the way rivalry was predicted by our variables of interest seemed to be restricted to sociodemographic characteristics.

Taken together, these results suggested that SZ-related indicators can help to explain sibling relationships on the conflict dimension, but not on the warmth or rivalry dimensions. We therefore inferred that the quality of the relationship between a healthy individual and a sibling with SZ is better predicted by other variables, such as those related to SZ.

#### Did SZ-Related Variables Explain Sibling Relationships in the SZ Sibling Group?

##### Clinical Data About the Sibling Diagnosed With SZ (SZ Sibling group n = 201)

Descriptive statistics for the SZ-related variables are displayed in **Table 4**. They were computed for the initial SZ sibling group (*N* = 201). At this descriptive level, we found that the majority (61%) of participants did not know which type of SZ their ill sibling had. SZ severity was reported by participants to be moderately high (*M* = 4.76 on a scale ranging from 1 = *not at all* to 7 = *very high*). Nevertheless, the siblings with SZ were described as having some contact with healthcare professionals. More specifically, 89% of them were declared to be receiving one form of treatment or another, and mean frequency of treatment attendance was 4.74. On the scale we used here, this corresponded to approximately one consultation with a healthcare professional every 2 weeks. Finally, only 13% of our participants described themselves as their sibling’s main caregiver.

**Table 4:** Descriptive statistics for the SZ sibling group (n = 201): Clinical data about the target sibling with SZ.

Variable	Modality (for categorical variables)	Mean
Mean (*SD*) age in years at symptom onset		18.81 (5.42)
Mean (*SD*) treatment attendance frequency, ranging from 1 (*daily*) to 7 (*never*)		4.74 (2.16)
Mean (*SD*) severity, ranging from 1 (*not at all serious*) to 7 (*extremely serious*)		4.76 (1.47)
		OR %
Mean number (*SD*) of hospitalizations		1.96 (1.08)
Proportion of participants who did not know which type of SZ their sibling had (%)	Not known	123 (61.19)
Presence of treatment (%)	With treatment	178 (88.56)
Caregiver identity (%)	Participant	28 (13.93)

##### Effect of Each SZ-Related Variable on the Quality of the Adult Sibling Relationship

To determine whether these SZ-related variables predicted the quality of the sibling relationship, we first examined the effect of each of these variables in turn (see **Table 5**). If a regression analysis simultaneously contained all our sociodemographic, family-structure, and SZ-related variables, the high number of predictor variables might weaken the effects of some of them. Results showed that symptom severity and not being treated were positively related to conflict (*β* = 0.28, *p* < 0.001, and *β* = 0.95, *p* < 0.001), whereas frequency of treatment attendance was negatively related to conflict (*β* = −0.17, *p* < 0.05).

**Table 5:** Standardized regression coefficients for the effects of each schizophrenia-related variable of interest on warmth, conflict, and rivalry for the SZ sibling group (n=201).

Variable	Compared modalities (for categorical variables)	Warmth	Conflict	Rivalry
Age at symptom onset in years		0.07	0.05	0.01
Proportion of participants who did not know which type of SZ their sibling had	Known vs. unknown	0.03	−0.02	0.12
Severity, ranging from 1 (*not at all serious*) to 7 (*extremely serious*)		−0.14	0.28***	0.05
Number of hospitalizations		0	0.07	0.08
Presence of treatment	Without vs. with	−0.32	0.95***	0.39
Frequency of treatment attendance, ranging from 1 (*daily*) to 7 (*never*)		0.02	−0.17*	−0.05
Caregiver identity	Other vs. participant	−0.37	−0.20	0.09

Values in the table are standardized regression coefficients.

*p < 0.05. **p < 0.01. ***p < 0.001.

##### Comparisons of Regression Models With or Without SZ-Related Variables Among Their Predictor Variables

Consistent with the initial results reported above, when a regression model (*n* = 201) containing solely the sociodemographic and family-structure variables that were significant predictors of the sibling relationship among SZ siblings (i.e., residence and visit frequency for warmth, residence for conflict, nothing for rivalry; see **Table 3**) and our SZ-related variables of interest among its predictor variables was compared with a model containing only sociodemographic and family-structure variables, it explained a significantly higher proportion (14% vs. 0%) of the variance for conflict, *F*(7, 190) = 5.38, *p* < 0.001 (see **Table 6**). Similar to the results observed when the SZ-related variables were considered one at a time, this increase in explained variance for conflict occurred when the effects of symptom severity (*β* = 0.22, *p* < 0.01), not being treated (*β* = 0.72, *p* < 0.001) and treatment attendance (*β* = 0.17, *p* < 0.05) were considered.

**Table 6:** Comparison of regression models of the three dimensions of the adult sibling relationship without and with schizophrenia-related variable.

	Model 1 *R²*	Model 2 *R²*	*Df*	*F*	*P*
Warmth	6.99	6.04	7, 189	0.66	0.72
Conflict	0	13.60	7, 190	5.38	<0.001
Rivalry	0	0	7, 173	0.82	0.62

“R²” refers to adjusted R². “Step 1” refers to regression analyses containing socio-demographic and family-structure predictor variables, whereas “Step 2” refers to regression analyses containing socio-demographic, family-structure socio-demographic and schizophrenia-related variable.

Taken together, these results suggested that SZ-related indicators can help to explain sibling relationships on the conflict dimension, but not on the warmth or rivalry dimensions.

## Discussion

The aim of the present study was to identify the variables that predict the three dimensions (warmth, conflict, and rivalry) of relationships between healthy adults and siblings with and without SZ. To this end, we ran regression analyses to compare the characteristics of two matched groups of adult participants with an ill or nonclinical sibling. In line with our hypotheses, results pointed to different sets of determinants of the sibling relationship, depending on whether the target sibling had been diagnosed with SZ. For nonclinical siblings, sociodemographic and family-structure variables helped to predict the experience of the adult sibling relationship, in terms of warmth, conflict, and rivalry. By contrast, when the target sibling had been diagnosed with SZ, these variables no longer sufficed to predict the healthy adult’s experience of the sibling relationship. Instead, results showed that we also needed to take account of SZ-related variables (age at symptom onset, severity and type of pathology, frequency of treatment, etc.) to explain a significantly greater proportion of the variance in the three dimensions. Including the participants’ perspective on different aspects of their sibling’s pathology did indeed help to explain their experience of the sibling relationship, but only on the conflict dimension.

### Comparison With Nonclinical Sibling Group

We begin by looking at each of the sociodemographic and family-structure variables we tested (socioeconomic status, participant’s sex, target sibling’s sex, age of participant, whether or not they lived under the same roof, and how often they saw each other), and assessing their ability to predict perceived warmth, conflict, and rivalry in the sibling relationship. For each of these variables, we interpret the results for the nonclinical sibling group and discuss why the variable generally did not explain any of the three dimensions in the SZ sibling group.

In the nonclinical sibling group, all three dimensions of the sibling relationship (warmth, conflict and rivalry) were partially explained by the participants’ *socioeconomic status*. Being unemployed had a mainly negative effect on the three dimensions of the sibling relationship, but only in the nonclinical group. The risks of isolation, and therefore of depression, were greater for the unemployed than for those who were in employment (58). By contrast, when the target sibling had been diagnosed with SZ, this variable socioeconomic status did not explain any of the variance in the three dimensions.

Still concerning nonclinical siblings, we found that *participant’s sex* only influenced rivalry. This result was partly in line with the literature, as women report greater rivalry in their sibling relationships than men do (15). However, the literature shows that sex also habitually influences the other two dimensions of adult sibling relationships (15, 59), which was not the case in our study. Rivalry was not influenced by participant’s sex in the SZ sibling group. This can be explained by the fact that sisters generally take on a caregiving role when their parents can no longer shoulder the burden, and caregiving seems not to leave any room for competition for parental affection.

As for the *sex of the target sibling*, our results showed that it influenced the warmth dimension, but only for nonclinical sibling group. There seemed to be less warmth when the target sibling was a man rather than a woman. Once again, these results tally with literature findings (15). By contrast, the sex of a target sibling with SZ had no effect on warmth. This result confirms Bowman et al.’s finding that sex had no effect on any of the three dimensions of adult sibling relationships when the target sibling had recently exhibited FEP symptoms (50).

Regarding *participant’s age*, results surprisingly showed that older participants with nonclinical siblings reported greater rivalry in their relationships, even though literature findings all indicate that these relationships grow less conflictual with age (18–21). Although this result is at odds with the literature, we can postulate that previous rivalries are reignited when a parent falls ill or dies (60). This does not happen in SZ sibling group, as the illness or death of a parent instead raises questions about who will take on the role of caregiver.

Analyses also revealed that *frequency of encounters* and *living under the same roof* were predictive of the warmth of sibling relationships in both groups. These results may seem only natural for nonclinical siblings, but are more surprising for SZ sibling group. Our results actually showed that more participants lived with the target sibling or went to visit them in the SZ sibling group than in the nonclinical sibling group. When they reach adulthood, children generally move away for work reasons and/or to start a family of their own (33). However, where a sibling has been diagnosed with SZ, they may feel they should not move away too far. In terms of the role they may play, some choose to support their ill sibling on a daily basis, acting very much as caregivers. In this case, they necessarily have more frequent contacts with their ill sibling, and may even share the same home. Whatever the case, our results underline the fact that geographical distance or proximity influences the experience of warmth in all sibling relationships.

In summary, the comparison of regression models indicated that sociodemographic and family-structure variables explained the three dimensions of sibling relationships far better when the target sibling did not have a mental disorder. Where this was the case, results showed that other variables specifically relating to that disorder explained the dimensions of the sibling relationship better.

### Addition of SZ-Related Variables

Nevertheless, the addition of SZ-related variables to the regression models only influenced participants’ experience of conflict-the dominant dimension in these particular sibling relationships (61). More specifically, our results showed that frequency of treatment and, above all, symptom severity were the best predictors of conflict in SZ sibling group.

In other words, for SZ sibling group, our results indicated that the conflict dimension was fueled by symptom severity. Healthy brothers and sisters rank delusions, poor personal hygiene, low treatment adherence, social isolation, lack of motivation, and verbal violence as the most disturbing symptoms (62). Moreover, a lack of information about their sibling’s disease can lead them to have misconceptions about SZ, such as thinking that their ill sibling can control and master his or her pathological behaviors (e.g., “He deliberately behaves strangely so that people think he’s eccentric”, “He thinks everyone’s spying on him as if he were someone important”). These misconceptions can engender hostile reactions. For instance, Smith and Greenberg explained that if healthy adults are laboring under the misapprehension that their ill sibling can control the pathological behaviors, this can harm the sibling relationship (51). In contrast to our results, however, these authors failed to observe an impact of symptom severity on the relationship (51). These divergent results can probably be explained by the fact that we collected participants’ subjective views of the severity of their sibling’s disease, whereas Smith and Greenberg administered an objectified scale (i.e., symptom scale from the Schizophrenia Outcome Module) to the primary caregivers. We can thus conclude that it is the subjective feeling rather than the objective assessment of disease severity that predicts perceived conflict in these sibling relationships.

Our results also indicated that conflict was explained better when frequency of treatment was taken into account. In other words, irregular follow up is predictive of what healthy adults perceive to be a more conflictual sibling relationship. Neglecting to attend regular follow-up consultations is one of the characteristic symptoms of SZ (63). It can be attributed to a lack of insight (63), in that if patients are not fully aware of all their symptoms, they may not adhere as rigorously to their treatment (64). As a consequence, they put less time and effort in their relationships with healthcare professionals, resulting in an intensification of their symptoms. In other words, frequency of treatment is linked to symptom severity, and each of these variables helps to explain conflict in SZ sibling relationships.

### Limitations

The present study shed light on the factors that contribute to the quality of the relationship between a healthy adult and a sibling with SZ. Nevertheless, it had several limitations, starting with the unbalanced sex ratio. Female overrepresentation is a recurrent feature of adult sibling studies (15, 65), and ours was no exception. It is, however, worth pointing out that Bowman et al. did not find any effect of sex on the three dimensions of sibling relationships, measured with the validated long version of the ASRQ (50), so it is unlikely that a more balanced sex ratio would have changed our results. In addition, the participants in our research mostly responded about a sick brother. This result is not surprising, given the high male/female ratio in SZ (66).

Second, there were biases in the way we matched the two groups. Whereas participants who had a sibling with SZ were asked to consider their relationship with that sibling when responding to the ASRQ-S, participants with nonclinical siblings could respond about the sibling who was closest to them in age. Although our participants were matched on more variables than those in previous studies comparing groups on sibling relationships (67–69), we did not take into account either age differences between the members of the dyads or the number of brothers and sisters, both variables liable to influence the dimensions of sibling relationships (54).

Finally, although several variables were found to influence the warmth and rivalry dimensions, more investigations must be undertaken in order to achieve a better understanding of these two dimensions of the sibling relationship when a brother or sister has SZ. To take these results further, more variables (e.g., number of people at home, dependent children, education level, etc.) should be included in the sociodemographic category. More family structure variables (e.g., parents’ marital status) should also be added to the regression model. In addition, considering a larger number of SZ-related variables would help to support these initial results (e.g., history of the sibling’s disease or objective presence of behavioral disorders).

### Perspectives and Conclusion

Our results therefore allowed us to identify different determinants of the sibling relationship, depending on whether the target sibling had been diagnosed with SZ. Sociodemographic and family-structure variables played a major role in explaining the three dimensions for nonclinical sibling relationships. By contrast, they had no explanatory value for the experience of SZ sibling relationships, where the level of conflict was mainly predicted by symptom severity and frequency of treatment. Our results therefore suggest that the main cause of poor relationships with a sibling diagnosed with SZ is symptom severity. These symptoms can be misinterpreted by the healthy brother or sister and trigger conflict. Despite the expansion in recent years of support and psychoeducational programs for the relatives of individuals with mental disorders, most of these interventions target parents and neglect siblings (70). It is therefore important to extend provision so that they, too, can be given information about the pathology and greater access to professionals. This will improve their understanding of SZ symptoms and thereby help them to cope with having an ill sibling. Moreover, enhancing the relationship that nonclinical individuals have with an ill sibling can improve the wellbeing of the former and by so doing, that of the latter. There is therefore a twofold benefit. It would thus be worthwhile exploring the possible interdependence between actors and partners in psychological wellbeing in future SZ dyadic studies.

## Data Availability Statement

The datasets generated for this study are available on request to the corresponding author.

## Ethics Statement

The studies involving human participants were reviewed and approved by Lille University’s ethics committee for human-based research (2018 – 276-S61). The patients/participants provided their written informed consent to participate in this study.

## Author Contributions 

HW and LP designed the study and edited and finalized the manuscript. LP acquired the data from the siblings with and without schizophrenia. LP and J-BP analyzed the data. HW, LP, and J-BP interpreted the data. LP wrote the first draft of the manuscript. All the authors reviewed (HW, J-BP, and EB), amended and approved the manuscript.

## Conflict of Interest

The authors declare that the research was conducted in the absence of any commercial or financial relationships that could be construed as a potential conflict of interest.

## Footnotes

1 National Union of Families or Friends of Persons with Mental Illness and/or Disability.

## References

[1] Lamb HR Bachrach LL Some perspectives on deinstitutionalization. Psychiatr Serv (2001) 52(8):1039–45. 10.1176/appi.ps.52.8.1039 11474048

[2] Wright ER Avirappattu G Lafuze JE The family experience of deinstitutionalization: insights from the closing of Central State Hospital. J Behav Health Serv Res (1999) 26(3):289–304. 10.1007/BF02287274 10425867

[3] Awad AG Voruganti LNP The burden of schizophrenia on caregivers: a review. PharmacoEconomics (2008) 26(2):149–62. 10.1016/j.apnu.2011.03.008 18198934

[4] Gutiérrez-Maldonado J Caqueo-Urízar A Kavanagh DJ Burden of care and general health in families of patients with schizophrenia. Soc Psychiatry Psychiatr Epidemiol (2005) 40(11):899–904. 10.1007/s00127-005-0963-5 16245190

[5] Pilling S Bebbington P Kuipers E Garety P Geddes J Orbach G Psychological treatments in schizophrenia: I. Meta-analysis of family intervention and cognitive behaviour therapy. psychol Med (2002) 32(5):763–82. 10.1017/S0033291702005895 12171372

[6] Sin J Gillard S Spain D Cornelius V Chen T Henderson C Effectiveness of psychoeducational interventions for family carers of people with psychosis: a systematic review and meta-analysis. Clin Psychol Rev (2017) 56:13–24. 10.1016/j.cpr.2017.05.002 28578249

[7] Winefield HR Harvey EJ Needs of family caregivers in chronic schizophrenia. Schizophr Bull (1994) 20(3):557–66. 10.1093/schbul/20.3.557 7973471

[8] Magliano L Fadden G Fiorillo A Malangone C Sorrentino D Robinson A Family burden and coping strategies in schizophrenia: are key relatives really different to other relatives? Acta Psychiatrica Scand (1999) 99(1):10–5. 10.1111/j.1600-0447.1999.tb05379.x 10066002

[9] Davtian H Scelles R Penser la formation des familles et de l’entourage d’une personne atteinte de schizophrénie. Annales Médico-psychologiques Rev Psychiatrique (2014) 172(9):735–40. 10.1016/j.amp.2013.06.017

[10] McHale SM Updegraff KA Whiteman SD Sibling relationships and influences in childhood and adolescence. J Marriage Family (2012) 74(5):913–30. 10.1111/j.1741-3737.2012.01011.x PMC3956653 24653527

[11] Gold DT Sibling relationships in old age: A typology. Int J Aging Hum Dev (1989) 28(1):37–51. 10.2190/VGYX-BRHN-J51V-0V39 2707894

[12] Von Benedek L Frères et sœurs pour la vie: l"empreinte de la fratrie sur nos relations adultes. Paris: Eyrolles (2013).

[13] Cicirelli VG Sibling relationships across the life span. (Boston, MA: Springer US) (1995).

[14] Milevsky A Smoot K Leh M Ruppe A Familial and contextual variables and the nature of sibling relationships in emerging adulthood. Marriage Family Rev (2005) 37(4):123–41. 10.1300/J002v37n04_07

[15] Stocker CM Lanthier RP Furman W Sibling relationships in early adulthood. J Family Psychol (1997) 11(2):210. 10.1037/0893-3200.11

[16] Buist KL Deković M Prinzie P Sibling relationship quality and psychopathology of children and adolescents: a meta-analysis. Clin Psychol Rev (2013) 33(1):97–106. 10.1016/j.cpr.2012.10.007 23159327

[17] Gass K Jenkins J Dunn J Are sibling relationships protective? A longitudinal study. J Child Psychol Psychiatry (2007) 48(2):167–75. 10.1111/j.1469-7610.2006.01699.x 17300555

[18] Pulakos J Brothers and sisters: nature and importance of the adult bond. J Psychol (1987) 121(5):521–2. 10.1080/00223980.1987.9915506

[19] Stewart RB Kozak AL Tingley LM Goddard JM Blake EM Cassel WA Adult sibling relationships: validation of a typology. Pers Relat (2001) 8(3):299–324. 10.1111/j.1475-6811.2001.tb00042.x

[20] White L Sibling relationships over the life course: a panel analysis. J Marriage Family (2001) 63(2):555. 10.1111/j.1741-3737.2001.00555.x

[21] White LK Riedmann A Ties among adult siblings. Soc Forces (1992) 71(1):85–102. 10.1093/sf/71.1.85

[22] Wu K Kim JHJ Nagata DK Kim SI Perceptions of sibling relationships and birth order among Asian American and European American emerging adults. J Family Issues (2018) 39(13):3641–63. 10.1177/0192513X18783465 PMC6426140 30906094

[23] Cicirelli VG The effect of sibling relationship on concept learning of young children taught by child-teachers. Child Dev (1972) 43(1):282. 10.2307/1127894 5027668

[24] Spitze G Trent K Gender differences in adult sibling relations in two-child families. J Marriage Family (2006) 68(4):977–92. 10.1111/j.1741-3737.2006.00308.x

[25] Tucker CJ Barber BL Eccles JS Advice about life plans and personal problems in late adolescent sibling relationships. J Youth Adolescence (1997) 26(1):63–76. 10.1023/A:1024540228946

[26] Lee TR Mancini JA Maxwell JW Sibling relationships in adulthood: contact patterns and motivations. J Marriage Family (1990) 52(2):431–40. 10.2307/353037

[27] Bleske-Rechek A Kelley JA Birth order and personality: a within-family test using independent self-reports from both firstborn and laterborn siblings. Pers Individ Dif (2014) 56:15–8. 10.1016/j.paid.2013.08.011

[28] Hughes C White N Birth order and sibling relationships. In: Shackelford TK Weekes-Shackelford VA, editors. Encyclopedia of Evolutionary Psychological Science. Cham: Springer International Publishing (2018). p. 1–10. 10.1007/978-3-319-16999-6_836-1

[29] Shumaker DM Miller C Ortiz C Deutsch R The forgotten bonds: the assessment and contemplation of sibling attachment in divorce and parental separation. Family Court Rev (2011) 49(1):46–58. 10.1111/j.1744-1617.2010.01352.x

[30] Cicirelli VG Effects of sibling structure and interaction on children’s categorization style. Dev Psychol (1973) 9(1):132. 10.1037/h0035061

[31] Cicirelli VG Relationship of sibling structure and interaction to younger sib’s conceptual style. J Genet Psychol (1974) 125(2):37–49. 10.1080/00221325.1974.10532301

[32] Pike A Coldwell J Dunn JF Sibling relationships in early/middle childhood: links with individual adjustment. J Family Psychol (2005) 19(4):523. 10.1037/0893-3200.19.4.523 16402867

[33] Conger KJ Little WM Sibling relationships during the transition to adulthood: young adult siblings. Child Dev Perspect (2010) 4(2):87–94. 10.1111/j.1750-8606.2010.00123.x 20700389 PMC2917987

[34] Riggio HR Structural features of sibling dyads and attitudes toward sibling relationships in young adulthood. J Family Issues (2006) 27(9):1233–54. 10.1177/0192513X06289103

[35] Sin J Moone N Harris P Siblings of individuals with first-episode psychosis. J Psychosoc Nurs Ment Health Serv (2008) 46(6):33–40. 10.3928/02793695-20080601-11 18595457

[36] Sin J Moone N Harris P Scully E Wellman N Understanding the experiences and service needs of siblings of individuals with first-episode psychosis: a phenomenological study. Early Intervention Psychiatry (2012) 6(1):53–9. 10.1111/j.1751-7893.2011.00300.x 21952020

[37] Riebschleger JL Families of chronically mentally ill people: siblings speak to social workers. Health Soc Work (1991) 16(2):94–103. 10.1093/hsw/16.2.94 2071070

[38] Racamier P-C Le paradoxe des Schizophrènes. Paris: PUF (1978).

[39] Kate N Grover S Kulhara P Nehra R Relationship of quality of life with coping and burden in primary caregivers of patients with schizophrenia. Int J Soc Psychiatry (2014) 60(2):107–16. 10.1177/0020764012467598 23292614

[40] Davtian H Collombet É Aidant familial en psychiatrie, une place “naturelle”? Empan (2014) 94(2):47–52. 10.3917/empa.094.0047

[41] Friedrich RM Lively S Rubenstein LM Siblings’ coping strategies and mental health services: a national study of siblings of persons with schizophrenia. Psychiatr Serv (2008) 59(3):261–7. 10.1176/appi.ps.59.3.261 18308906

[42] Gerace LM Camilleri D Ayres L Sibling perspectives on schizophrenia and the family. Schizophr Bull (1993) 19(3):637–47. 10.1093/schbul/19.3 8235464

[43] Stålberg G Ekerwald H Hultman CM At issue: siblings of patients with schizophrenia: sibling bond, coping patterns, and fear of possible schizophrenia heredity. Schizophr Bull (2004) 30(2):445–58. 10.1093/oxfordjournals.schbul.a007091 15279059

[44] Hatfield AB Lefley HP Future involvement of siblings in the lives of persons with mental illness. Community Ment Health J (2005) 41(3):327–38. 10.1007/s10597-005-5005-y 16131010

[45] Horwitz AV Siblings as caregivers for the seriously mentally ill. Milbank Q (1993) 71(2):323–39. 10.2307/3350402 8510604

[46] Horwitz AV Predictors of adult sibling social support for the seriously mentally ill: an exploratory study. J Family Issues (1994) 15(2):272–89. 10.1177/0192513X94015002007

[47] Lohrer SP Lukens EP Thorning H Economic expenditures associated with instrumental caregiving roles of adult siblings of persons with severe mental illness. Community Ment Health J (2006) 43(2):129–51. 10.1007/s10597-005-9026-3 16514475

[48] Schmid R Schielein T Binder H Hajak G Spiessl H The forgotten caregivers: siblings of schizophrenic patients. Int J Psychiatry Clin Pract (2009) 13(4):326–37. 10.3109/13651500903141400 24916945

[49] Smith MJ Greenberg JS Mailick Seltzer M Siblings of adults with schizophrenia: expectations about future caregiving roles. Am J Orthopsychiatry (2007) 77(1):29–37. 10.1037/0002-9432.77.1.29 17352582 PMC2396553

[50] Bowman S Alvarez-Jimenez M Howie L McGorry P Wade D The impact of first-episode psychosis on the sibling relationship. Psychiatry (2015) 78(2):141–55. 10.1080/00332747.2015.1051444 26168092

[51] Smith MJ Greenberg JS Factors contributing to the quality of sibling relationships for adults with schizophrenia. Psychiatr Serv (2008) 59(1):57–62. 10.1176/ps.2008.59.1.57 18182540 PMC2396577

[52] Janghorban R Roudsari RL Taghipour A Skype interviewing: the new generation of online synchronous interview in qualitative research. Int J Qual Stud Health Well-being (2014) 9(1):24152. 10.3402/qhw.v9.24152 24746247 PMC3991833

[53] World Medical Association World Medical Association Declaration of Helsinki: ethical principles for medical research involving human subjects. JAMA (2013) 310(20):2191–4. 10.1001/jama.2013.281053 24141714

[54] Furman W Buhrmester D Children’s perceptions of the qualities of sibling relationships. Child Dev (1985) 56(2):448–61. 10.2307/1129733 3987418

[55] Vallieres EF Vallerand RJ Traduction et validation canadienne-française de l’Echelle de l’Estime de Soi de Rosenberg. Int J Psychol (1990) 25(2):305–16. 10.1080/00207599008247865

[56] Fox J Weisberg S An R companion to applied regression. SAGE publications (2018).

[57] James G Witten D Hastie T Tibshirani R An introduction to statistical learning: with applications in R. New York: Springer-Verlag; (2013).

[58] Herbig B Dragano N Angerer P Health in the long-term unemployed. Deutsches Aerzteblatt Int (2013) 110(23/24):413–9. 10.3238/arztebl.2013.0413 PMC3702026 23837086

[59] Buhrmester D Furman W Perceptions of sibling relationships during middle childhood and adolescence. Child Dev (1990) 61(5):1387–98. 10.1111/j.1467-8624.1990.tb02869.x 2245732

[60] Lamb ME Sutton-Smith B Sibling relationships: their nature and significance across the lifespan. Psychology Press (2014).

[61] Plessis L Wilquin H Pavani J-B Bouteyre E Comparison of relationships among French adult siblings with or without schizophrenia using the ASRQ-S: mediating effect on emotional distress. BMC Psychiatry (2020) 20122. 10.1186/s12888-020-02510-6 PMC7071713 32169060

[62] Lively S Friedrich RM Rubenstein L The effect of disturbing illness behaviors on siblings of persons with schizophrenia. J Am Psychiatr Nurses Assoc (2004) 10(5):222–32. 10.1177/1078390304269497

[63] Béland S Lepage M The relative contributions of social cognition and self-reflectiveness to clinical insight in enduring schizophrenia. Psychiatry Res (2017) 258:116–23. 10.1016/j.psychres.2017.09.082 28992548

[64] Rüsch N Corrigan PW Motivational interviewing to improve insight and treatment adherence in schizophrenia. Psychiatr Rehabil J (2002) 26(1):23–32. 10.2975/26.2002.23.32 12171279

[65] Walęcka-Matyja K Psychometric properties of the Polish adaptation of the Adult Sibling Relationship Questionnaire (ASRQ). Arch Psychiatry Psychother (2014) 16(4):77–88. 10.12740/APP/32460

[66] Mendrek A Mancini-Marïe A Sex/gender differences in the brain and cognition in schizophrenia. Neurosci Biobehav Rev (2016) 67:57–78. 10.1016/j.neubiorev.2015.10.013 26743859

[67] O’Neill LP Murray LE Anxiety and depression symptomatology in adult siblings of individuals with different developmental disability diagnoses. Res Dev Disabil (2016) 51–52:116–25. 10.1016/j.ridd.2015.12.017 26820453

[68] Orsmond GI Seltzer MM Siblings of individuals with autism or Down syndrome: effects on adult lives. J Intellectual Disability Res (2007) 51(9):682–96. 10.1111/j.1365-2788.2007.00954.x 17845237

[69] Rimmerman A Involvement with and role perception toward adult siblings with and without mental retardation. J Rehabil (2001) 67(2):10.

[70] Young L Murata L McPherson C Jacob JD Vandyk AD Exploring the experiences of parent caregivers of adult children with schizophrenia: a systematic review. Arch Psychiatr Nurs (2018) 33(1):93–103. 10.1016/j.apnu.2018.08.005 30663631

